# Preparation of Copper Surface for the Synthesis of Single-Layer Graphene

**DOI:** 10.3390/nano11051071

**Published:** 2021-04-22

**Authors:** Ivan Kondrashov, Maxim Komlenok, Pavel Pivovarov, Sergey Savin, Elena Obraztsova, Maxim Rybin

**Affiliations:** 1 Prokhorov General Physics Institute of the Russian Academy of Sciences, 38 Vavilov St., 119991 Moscow, Russia; komlenok@nsc.gpi.ru (M.K.); p_pivovarov@hotmail.com (P.P.); elobr@mail.ru (E.O.); 2 MIREA—Russian Technological University, 78 Vernadsky Avenue, 119454 Moscow, Russia; savin@mirea.ru; 3 Moscow Institute of Physics and Technology, State University, 1 “A” Kerchenskaya St., 117303 Moscow, Russia

**Keywords:** CVD synthesis, graphene monolayer, surface treatment, electrochemical polishing

## Abstract

Chemical vapor deposition synthesis of graphene on copper foil from methane is the most promising technology for industrial production. However, an important problem of the formation of the additional graphene layers during synthesis arises due to the strong roughness of the initial copper foil. In this paper, various approaches are demonstrated to form a smooth copper surface before graphene synthesis to reduce the amount of few layer graphene islands. Six methods of surface processing of copper foils are studied and the decrease of the roughness from 250 to as low as 80 nm is achieved. The correlation between foil roughness and the formation of the additional layer is demonstrated. Under optimized conditions of surface treatment, the content of the additional graphene layer drops from 9 to 2.1%. The quality and the number of layers of synthesized graphene are analyzed by Raman spectroscopy, scanning electron microscopy and measurements of charge mobility.

## 1. Introduction

Chemical vapor deposition (CVD) synthesis of graphene on metal substrates is the most promising method for the growing large-area films of high-quality [1]. Copper foil is usually chosen as a catalytic substrate for graphene synthesis since the single layer is easily grown due to the limited solubility of carbon in copper [2]. The quality of graphene depends not only on precursors and CVD parameters (pressure, temperature), but also on the properties of the copper substrate such as thickness, surface roughness, polycrystallinity, grain size, orientation and the presence of surface impurities [3,4,5,6,7]. In more detail, during the synthesis of graphene, methane decomposes into carbon and hydrogen near the catalytic copper foil and atomic carbon is deposited on the surface of copper. After the formation of a certain amount of carbon on the surface of the copper foil, nuclei are formed and their further growth results in the formation of a uniform film. These nuclei are more often found on surface irregularities and carbon atoms also accumulate on them. Thus, if the copper surface is well developed and has a strong roughness, then the formation of additional graphene layers during synthesis becomes more likely than on a smooth surface.

Copper foil is usually produced by mechanical rolling resulting in scratches along the rolling direction. As reported, the density and uniformity of graphene domains correlate with the surface roughness of the copper substrate [8,9]. One of the most efficient ways to grow an ideal graphene monolayer is to use a smooth single-crystal copper [10]. However, this approach is technologically difficult to implement and scale, since it requires the purchase of expensive single-crystal copper or recrystallization of a copper film. A simpler and more affordable option to reduce the formation of the multilayer graphene is to reduce surface roughness by processing the surface of copper before the synthesis. Different pretreatment methods have been investigated such as physical polishing [11], chemical etching [12,13], annealing [10,14,15,16], resolidification [17] and electrochemical polishing (ECP) [18,19,20,21,22,23] to smooth the surface. Mechanical polishing is a good method to get a smooth surface, but a thick copper substrate and the use of a special grinding machine are needed. Chemical treatment is effective for cleaning the surface of copper from impurities, but it is necessary to adjust the concentration of strong etchants based on nitric acid and the exposure time to avoid complete etching of copper. Annealing (resolidification) is a relatively simple method for smoothing the surface of copper. It is based on a rearrangement of surface copper atoms, which leads to the release of internal stresses and an increase in crystal size. However, this approach requires a high temperature close to the melting point of copper (1085 °C) and a long time for processing. ECP is the most effective method of cleaning and smoothing the surface of copper foil, which takes a few minutes and requires only a phosphoric acid and a power supply not exceeding 10 Watts.

In this work, we compare several methods for smoothing the foil before graphene synthesis, such as etching in nitric acid, ECP in phosphoric acid, long-term annealing in a hydrogen atmosphere for 5 h, as well as a combination of these methods. We investigate the surface roughness of copper foils after various treatments and then grow a graphene film on each type of prepared surface under the same conditions. We analyze the formation of additional graphene layers only inside one copper grain, regardless of its size and orientation. The dependence of the formation of the additional and following graphene layers on the roughness parameter of the prepared foils is established and the optimal conditions of pretreatment are determined. We also compare graphene synthesized on treated copper foil and the smooth surface of a copper film, deposited on a polished sapphire substrate.

## 2. Materials and Methods

We developed a protocol for the investigation of the effect of copper treatment on graphene formation. Before synthesis, the roughness of copper foil was analyzed by interference profilometry. All graphene samples were synthesized on copper foil using the procedure described in Section 2.3. The synthesized graphene samples were first studied using scanning electron microscopy and then transferred onto silicon wafers with SiO_2_ 90 nm or 300 nm thickness for best contrast in the optical microscope described in Section 2.4. Raman spectroscopy was used to analyze the quality of graphene and the number of graphene layers.

### 2.1. Pretreatment of Copper Substrates

Alfa Aesar copper foil (25 μm, 99.8%) was used for various processing methods before the graphene synthesis. The chemical etching of the sample in nitric acid (65% concentration) was performed in solution with a concentration of H_2_O:HNO_3_= 2:1 for 60 s. ECP was made using copper foil as anode placed in parallel to bulk copper substrate cathode with an interelectrode spacing of 2 cm in phosphoric acid (85% concentration) for 90 s at room temperature. Large irregularities dissolve first due to the effect of electric current. The annealing of the copper sample was performed at 1000 °C in a hydrogen atmosphere under 100 mbar for 5 h.

### 2.2. Copper Film Deposition

The copper film was deposited on a single-crystal sapphire substrate in a UNIVEX 300 vacuum chamber at a pressure of 10^−5^ mbar by passing an electric current through a molybdenum boat with copper metal pieces. The molybdenum boat was heated up to 1700–2000 °C and the copper evaporated and deposited on the target substrate. The thickness of the copper film was 1000 nm and was determined by the mass of metal placed into the boat.

### 2.3. Graphene Growth Procedure

The synthesis of graphene on copper foil has been well studied and various scientific groups have presented numerous options for the synthesis of graphene on copper foil under various conditions. The review [24] presents the basic principles of the formation of graphene on copper. For each experimental setup, it is necessary to select the optimal parameters of the graphene synthesis process. In this work, we used a standard tube furnace and the main feature of the synthesis was the absence of gas flow. The following optimal parameters of graphene growth were determined in this configuration: pressure 100 mbar, the gas concentration of H_2_:CH_4_ = 100:1, temperature 1000 °C for 20 min. Before the graphene formation, the copper foil was annealed at 1000 °C for 30 min in a hydrogen atmosphere at 100 mbar to remove natural oxide from the copper foil. This pretreatment changes neither the roughness of the foil nor the average size of the crystallites.

### 2.4. Transfer of the Grown Graphene

After the growth, the graphene was transferred from the copper to a SiO_2_/Si substrate using standard “wet” transfer technology [25]. First, polymethylmethacrylate (PMMA, 4% in anisole) was applied to the copper foil by the spin-coating method at 3000 rpm for 45 s. Then, the copper was etched with ammonium persulfate ((NH_4_)_2_S_2_O_8_). Finally, a PMMA/graphene film was washed with DI water and transferred onto the target substrate and PMMA was removed with acetone.

### 2.5. Sample Characterization

The quality of graphene films and the number of islands with additional graphene layers were investigated using optical microscopy and Raman scattering. Raman spectra obtained with a 514 nm excitation wavelength using 25% of 20 mW laser. The electrical characteristics of the graphene films were obtained using the four-probe method and Hall effect measurements. The surface morphology of the copper foil was examined by a white-light interference microscope (ZYGO NewView 5000, ZYGO Corp., Middlefield, CT, USA) and a scanning electron microscope (TESCAN Mira 3, TESCAN ORSAY HOLDING, Brno, Czech Republic). The amount of the additional layer on the surface area was calculated using graphics software for image analysis of transferred graphene films obtained using an optical microscope. The second and subsequent graphene layers transferred from copper foil onto 90 nm or 300 nm SiO_2_/Si substrate had a higher contrast (darker areas) in optical images than the monolayer. This difference was enough to set the monolayer as the base contrast and calculate the number of all darker spots as a percentage of the entire surface area.

## 3. Results and Discussion

**Figure 1:**
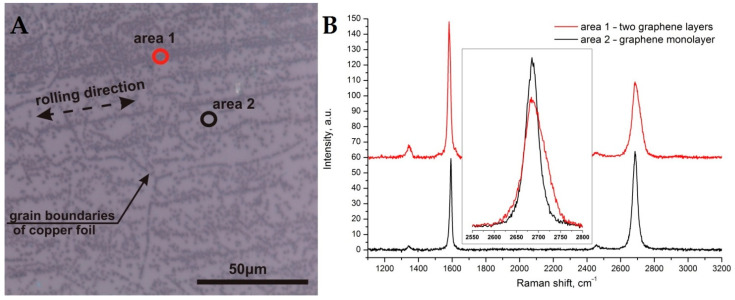
Optical image of graphene on Si/SiO_2_ 90 nm (**A**). Raman spectra of different graphene areas (**B**).

According to the described protocol at first, the graphene was synthesized on initial copper foil, transferred onto Si/SiO_2_ substrate and analyzed by Raman spectroscopy (see Figure 1). The picture clearly shows dark areas that correspond to the additional layer of graphene (area 1), as seen from the Raman spectra. Moreover, the optical contrast shows that the graphene film uniformly covers the entire area and the additional layer is covered according to a clear pattern. This pattern corresponds to the direction of rolling of copper foil during its manufacture. It is known that to make a thin foil (25 micrometers), a thick copper sheet is rolled several times between rolls to reduce the thickness. It is because of the unevenness of these shafts that furrows are formed on the copper foil. Other features of the appearance of additional layers of graphene are associated with irregularities of the polycrystalline foil at the junction of metal grains. Figure 1 (left part) clearly shows the lines of formation of additional graphene layers forming the boundaries of the foil crystallites. The Raman spectra of single-layer graphene and additional graphene layer are presented in Figure 1 (right part)and the comparison of 2D bands from different areas of samples is demonstrated in Figure 1 (right part) in the inset. The evolution of single-layer graphene to double-layer graphene is clearly seen [26].

In the introduction, the mechanism of graphene formation on the surface of a copper foil was described. Thus, to form a homogeneous graphene film with a minimum number of regions with additional graphene layers, it is necessary to smooth out all the irregularities of the copper foil.

**Figure 2:**
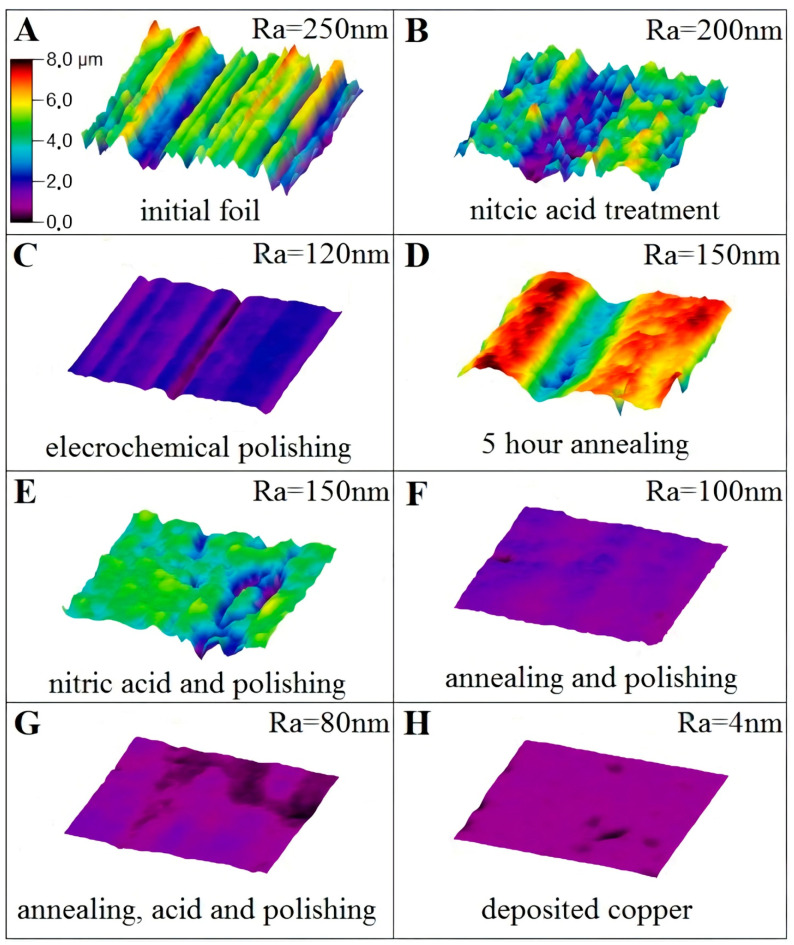
The roughness measurements of copper foil by interference microscope: (**A**)—without pre-treatment, (**B**)—nitric acid treatment, (**C**)—electrochemical polishing, (**D**)—hydrogen annealing for 5 h, (**E**)—nitric acid treatment and electrochemical polishing, (**F**)—hydrogen annealing and electrochemical polishing, (**G**)—hydrogen annealing, nitric acid treatment and electrochemical polishing, (**H**)—deposited copper film. All measurements were done froma 50 × 50 micrometers area. All figures have the same scale bar in color grade and it is indicated in Figure 2A.

In this work, six methods were used to form the surface with minimal roughness. The result of the analysis of surface morphology after different treatments is shown in Figure 2. The initial copper foil has the arithmetic average roughness Ra about 250 nm (Figure 2A). The treatment of the sample in nitric acid leads to Ra about 200 nm (Figure 2B), while ECP can provide Ra = 120 nm of the foil (Figure 2C). The annealing process results in the same Ra = 150 nm of copper foil as the combination of nitric acid treatment with further ECP, see Figure 2D,E, respectively. The combination of annealing and further polishing, as well as annealing and further acid treatment and ECP results in the roughness of 100 and 80 nm and are presented in Figure 2F,G, respectively. For comparison, the morphology of thin copper film (Ra = 4 nm) deposited on an optically flat single-crystal sapphire substrate is shown in Figure 2H.

**Figure 3:**
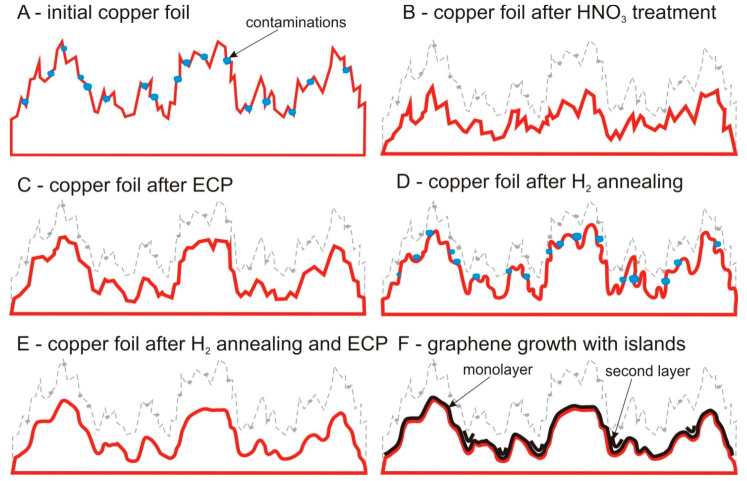
Principle scheme of a change in the copper surface roughness during various treatments (**A**–**E**) and formation of graphene layers (**F**).

The sketches shown in Figure 3, demonstrate the evolution of the copper surface under different processing methods (Figure 3A–E) and graphene growth (Figure 3F). The original sample has both deep grooves and small sharp irregularities from rolling, as well as various types of contaminants on the surface since industrial foil has a purity of no higher than 99.8% (Figure 3A). Using treatment with nitric acid, uniform etching occurs over the entire surface area of the foil, as well as an etching of impurities and contaminants from the surface. The foil steadily decreases in thickness and the surface topography remains almost unchanged (Figure 3B). The process of ECP consists of applying a potential difference between the initial copper foil and another electrode, which are in parallel placed in an electrolyte (phosphoric acid). In the electrolyte, a current begins to flow between the most strongly protruding irregularities of the foil, therefore, they are actively chemically etched and the copper foil is smoothed, but the deep grooves do not decrease (Figure 3C). Another approach to smoothing the foil is long-term annealing. We used annealing at temperature 1000 °C in a hydrogen atmosphere for 5 h. Such processing leads to the melting of small irregularities and smoothing of the foil, but all contaminants and impurities remain on the surface (Figure 3D). The most effective approach would be a combination of described methods, for example, ECP and annealing. In this case, all impurities and contaminants are removed during the polishing process and small protrusions are smoothed on the surface after long-term annealing (Figure 3E). In the case of a well-prepared copper foil, graphene film is formed on the entire surface and follows the foil relief, but carbon atoms accumulated during the decomposition of methane on the deepest irregularities result in the formation of nuclei of the additional layers (Figure 3F).

**Figure 4:**
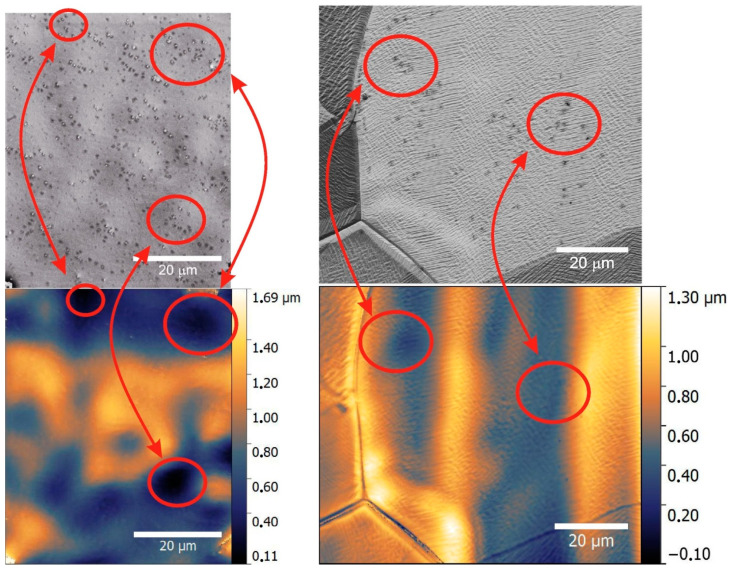
Upper row—SEM images of graphene films on copper foils. Low row—Interference profilometry images of the same areas of graphene film on copper foils. The left column corresponds to copper foil treated in nitric acid. The right column corresponds to copper foil treated by electrochemical polishing.

The next step in the research was to confirm the formation of additional graphene layers. The process of nucleation of the second layer of graphene and its correlationto the roughness of the copper surface is investigated using images obtained by an electron microscope and the images obtained by an interference profilometer from the same area. Figure 4 shows the results of comparing graphene films on foils after treatment in nitric acid (left column) and after electrochemical polishing (right column). The figure shows the regions of depressions on the foil, in which the second and subsequent graphene layers are formed. Moreover, after electrochemical polishing, the foil is smoother and the density of the formations of additional graphene layers is much lower. It should be noted that, in our experiments, the crystallographic orientation of copper does not affect the growth of additional graphene layers as strongly [27] as the roughness of copper.

**Figure 5:**
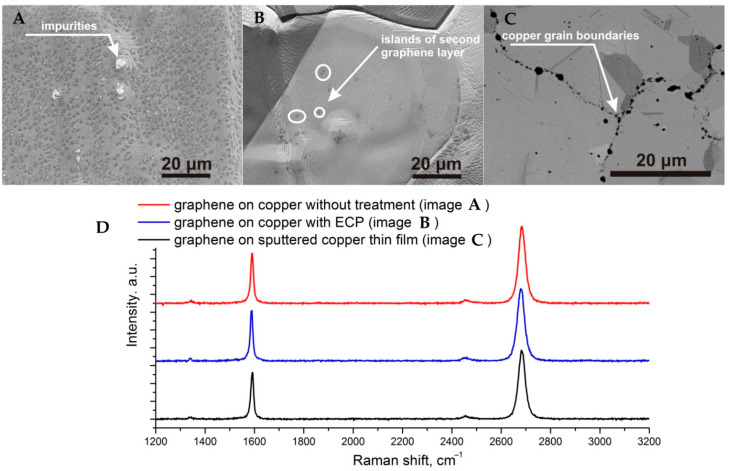
SEM images and Raman spectra (**D**) of graphene films synthesized on copper foils with different treatment ((**A**)—without pre-treatment, (**B**)—polishing) and (**C**)—deposited copper.

The analysis of obtained graphene films by Raman spectroscopy is presented in Figure 5D and the corresponded areas are demonstrated in Figure 5A–C. The SEM image of graphene film synthesized on initial copper foil without any processing is shown in Figure 5A. Almost 10% of the total area of initial copper foil is occupied by islands of dark gray color, which correspond to additional graphene layers. The copper foil treated by ECP with synthesized graphene which has the lowest density of the few layer islands is demonstrated in Figure 5B. Surface defects were removed by electropolishing and followed annealing of the copper surface led to smooth the surface more. A smoother copper surface provides lower nucleation sites for the graphene and as a result graphene film has less area of the additional layers. Despite the minimal roughness of the copper film, it is not possible to completely get rid of the additional layers, because they grow at the copper grain edges, unlike the irregularities and impurities in the case of copper foils. It should be also mentioned that most of the impurities on the surface of the copper film are located on the grain boundaries of neighboring crystallites. The Raman spectra in Figure 5D show that the quality of graphene films is the same for all substrates and does not depend on the surface processing of copper foils, the only difference is the number and average size of islands with few layer graphene film. Raman spectra from 30 points of each sample over the entire area of 10 *×* 10 mm were measured and there was no significant difference in the spectra. Measurements of the carrier mobility and sheet resistance, carried out by the four-probe method, have shown values from 2000 to 3000 cm^2^/(V·s) and less than 500 Ohm/square, respectively, that indicates the high quality of the synthesized graphene film.

**Figure 6:**
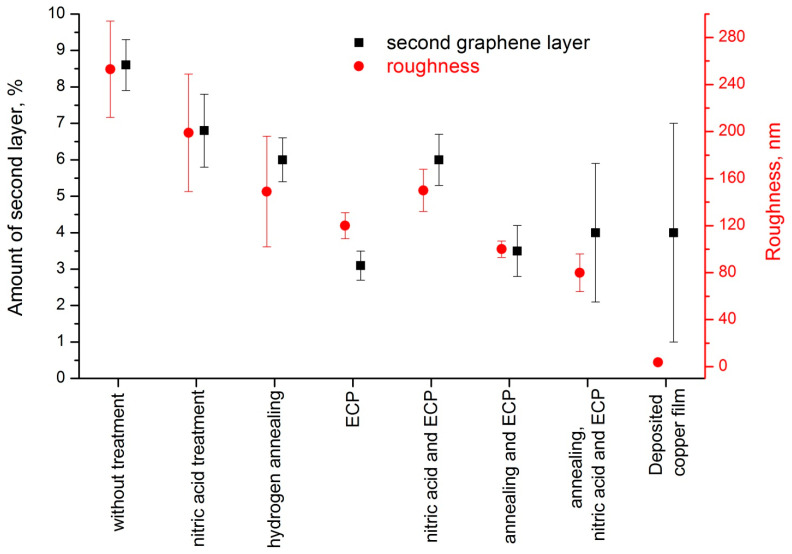
The effect of method of copper treatment on average roughness of copper surface (red circles) and the amount of graphene additional layer (black squares).

Five regions (100 × 100 μm^2^) of graphene transferred onto silicon oxide were taken from each sample and analyzed using optical microscopy. It was confirmed already that combination of the Raman analysis and the optical microscopy is enough for the identification of 1 and 2 layers of graphene [28,29]. A more detailed description of the calculation of the additional graphene layers in each sample is given in the Supplementary Information Figures S1–S6. The calculated percentage of two-layer graphene for all types of processing of copper foil is shown in Figure 6. The dependence of surface roughness on the type of treatment is presented in the same plot for comparison. It is seen that with a decrease in roughness, the amount of the additional layer of graphene also decreases. However, with a decrease in roughness below 120 nm, the amount of the additional layer remains approximately the same. This can be explained by a decrease in the number of the nucleus (islands) and an increase in the size of islands with a few layer graphene film. For example, for untreated foil (Ra = 250 nm), more than 1000 islands with an area of 6–9 μm^2^ are obtained in the area of 100 × 100 μm^2^, that is, the total area of the few layer graphene film (ρ) is 8.1 ÷ 9.4% of the entire surface (Figure S1). After the ECP processing (Ra = 120 nm), 150–200 islands are formed in the same region, but the size of each of them is reduced to 15–20 μm^2^ (ρ = 2.7–3.5%) (Figure S3). In the case of a combination of annealing, chemical etching and ECP (Ra = 80 nm), less than 50–80 islands with an area of 100 *×* 100 μm^2^ are formed on the foil, that corresponds to a percentage ρ = 2.1 ÷ 5.9% (Figure S7). On a deposited copper film with a roughness of only 4 nm, the formation of an additional graphene layer occurs at the grain boundary of copper crystals, but the size of islands can grow significantly. So only 10–20 islands of the two-layer graphene with an area of 50–100 μm^2^ are observed and their total area takes the same percentage ρ = 1 ÷ 7%.

## 4. Conclusions

The chemical vapor deposition technique is an efficient approach for the synthesis of high-quality graphene on copper foil over large areas. The roughness of the copper surface is the key parameter for the fabrication of the most uniform single-layer graphene film. The correlation between foil roughness and the formation of the additional layer is demonstrated. The use of the original commercial copper foil with Ra = 250 nm leads to the formation of a few layer graphene film on an area of 9% of the total surface. Additional processing of catalytic copper foil causes a decrease in the amount of the additional graphene layers. The lowest roughness of 80 nm is achieved using a combination of annealing, etching and electrochemical polishing, which results in the synthesis of the film with only 2.1% of the additional graphene layers. It is found that the optimal treatment is simply electrochemical polishing, which gives the same amount of a few layer graphene film, but on a copper surface with Ra = 120 nm. A further decrease in roughness even to 4 nm does not lead to a decrease in the amount of the additional graphene layers.

## Data Availability

The data presented in this study are available on request from the corresponding author.

## Supplementary Materials

The following are available online at https://www.mdpi.com/article/10.3390/nano11051071/s1, Information represent counting the number of additional graphene layers synthesized on copper foils processed in various ways. The amount of the additional layers on the surface area was calculated using graphics software for image analysis of transferred graphene films obtained using an optical microscope. The second and subsequent graphene layers had a higher contrast (darker areas) in optical images than the monolayer. This difference was enough to set the monolayer as the base contrast and calculate the number of all darker spots as a percentage of the entire surface area.
