# Supplementary material for: Preparation of Copper Surface for the Synthesis of Single-Layer Graphene
Source: Nanomaterials (Basel). 2021 Apr 22;11(5):1071. doi: 10.3390/nano11051071 (PMC8143535; doi:10.3390/nano11051071)
Supplement: Supplementary file 1 [file nanomaterials-11-01071-s001.zip › nanomaterials-1171482-supplementary.pdf]

nanomaterials

Supporting Information

Article

Preparation of Copper Surface for the Synthesis of Single-Layer Graphene

Ivan Kondrashov[1,*], Maxim Komlenok[1], Pavel Pivovarov[1], Sergey Savin[2], Elena Obraztsova[1,3] and Maxim Rybin[1,*]

1 Prokhorov General Physics Institute of the Russian Academy of Sciences, 38 Vavilov St., 119991 Moscow, Russia; komlenok@nsc.gpi.ru (M.K.); p_pivovarov@hotmail.com (P.P.); elobr@mail.ru (E.O.)
2 MIREA—Russian Technological University, 78 Vernadsky Avenue, 119454 Moscow, Russia; savin@mirea.ru
3 Moscow Institute of Physics and Technology, State University, 1 "A" Kerchenskaya St., 117303 Moscow, Russia
* Correspondence: navi.soul@gmail.com (I.K.); rybmaxim@gmail.com (M.R.)

Counting the number of additional graphene layers synthesized on copper foils processed in various ways.The amount of the additional layer on the surface area was calculated using graphics software for image analysis of transferred graphene films obtained using an optical microscope. The second and subsequent graphene layers transferred from copper foil onto 300 nm SiO_2/Si substrate had a higher contrast (darker areas) in optical images than the monolayer. This difference was enough to set the monolayer as the base contrast and calculate the number of all darker spots as a percentage of the entire surface area.

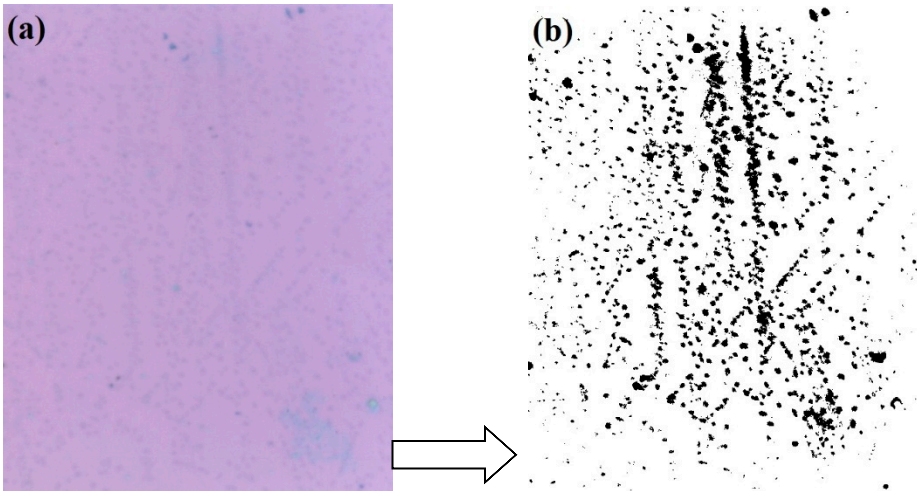

Figure S1. Optical image of graphene film transferred onto $SiO_2$300 nm after synthesis on initial copper foil (**a**). Image translation into two-color format for counting the number of additional graphene layers (**b**). The amount of second graphene layer is 8.6%.

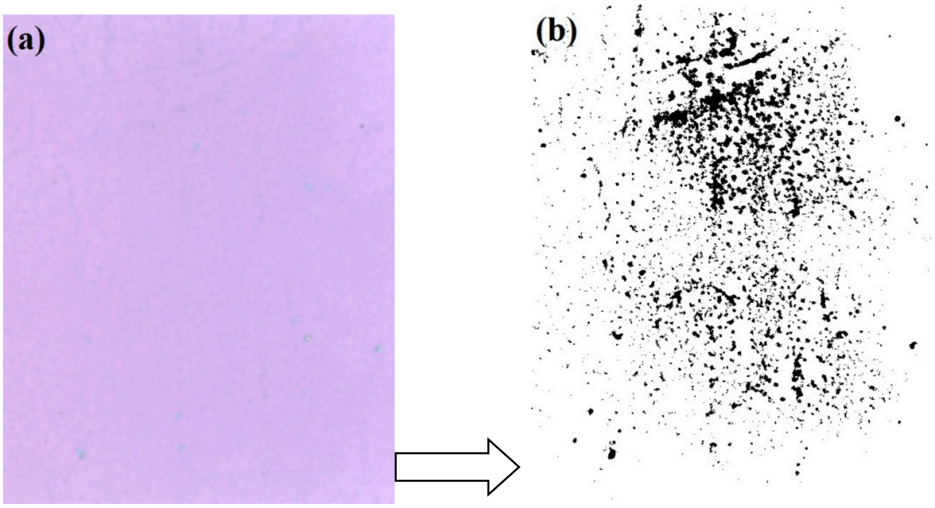

Figure S2. Optical image of graphene film transferred onto SiO_2 300 nm after synthesis on copper foil with nitric acid treatment (**a**). Image translation into two-color format for counting the number of additional graphene layers (**b**). The amount of Scheme 6. 8%.

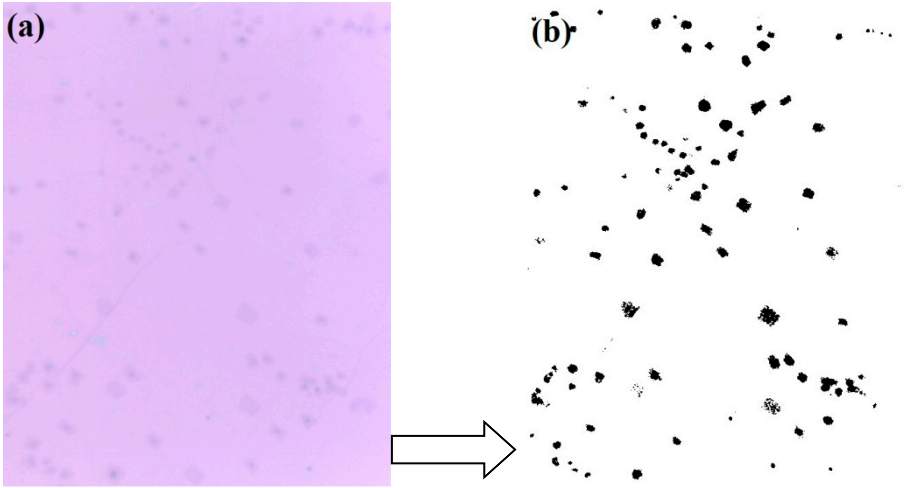

Figure S3. Optical image of graphene film transferred onto $SiO_2$300 nm after synthesis on copper foil with electropolishing treatment (**a**). Image translation into two-color format for counting the number of additional graphene layers (**b**). The amount of second graphene layer is 3.1%.

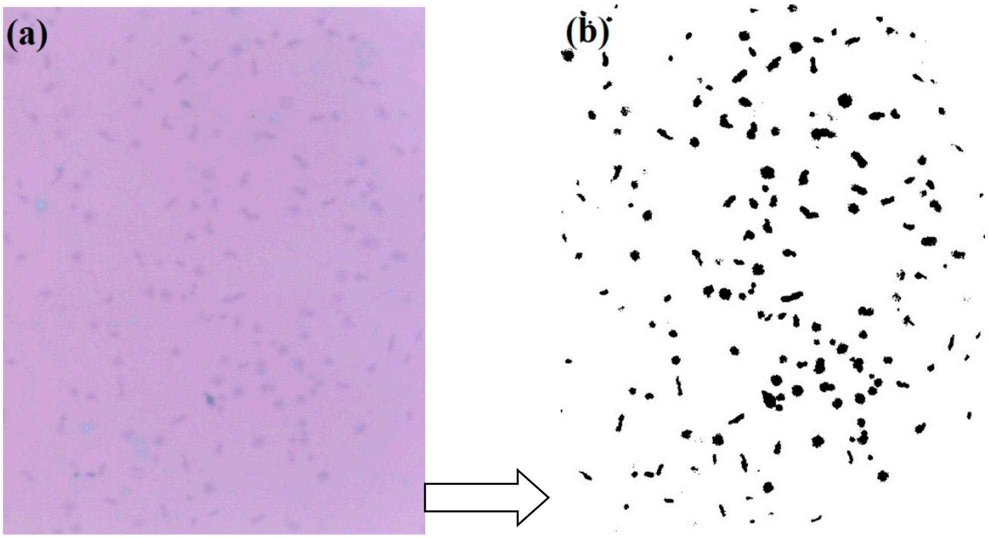

Figure S4. Optical image of graphene film transferred onto $SiO_2$300 nm after synthesis on copper foil with nitric acid and electropolishing treatment (**a**). Image translation into two-color format for counting the number of additional graphene layers (**b**). The amount of second graphene layer is 6.0%.

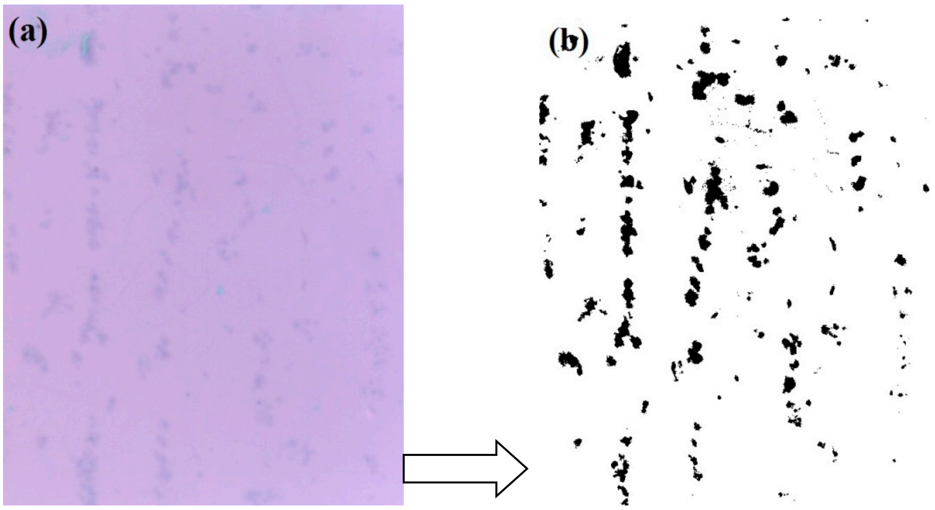

Figure S5. Optical image of graphene film transferred onto $SiO_2$300 nm after synthesis on copper foil with 5 hours hydrogen annealing (**a**). Image translation into two-color format for counting the number of additional graphene layers (**b**). The amount of second graphene layer is 6.0%.

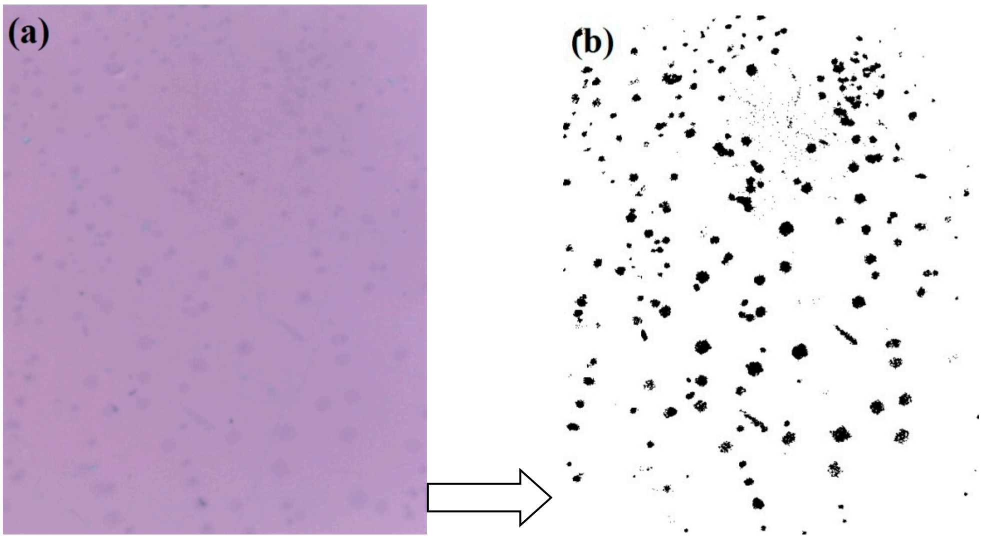

Figure 6. Optical image of graphene film transferred onto $SiO_2$300 nm after synthesis on copper foil with 5 hours hydrogen annealing and electrochemical polishing (**a**). Image translation into two-color format for counting the number of additional graphene layers (**b**). The amount of second graphene layer is 3.5%.

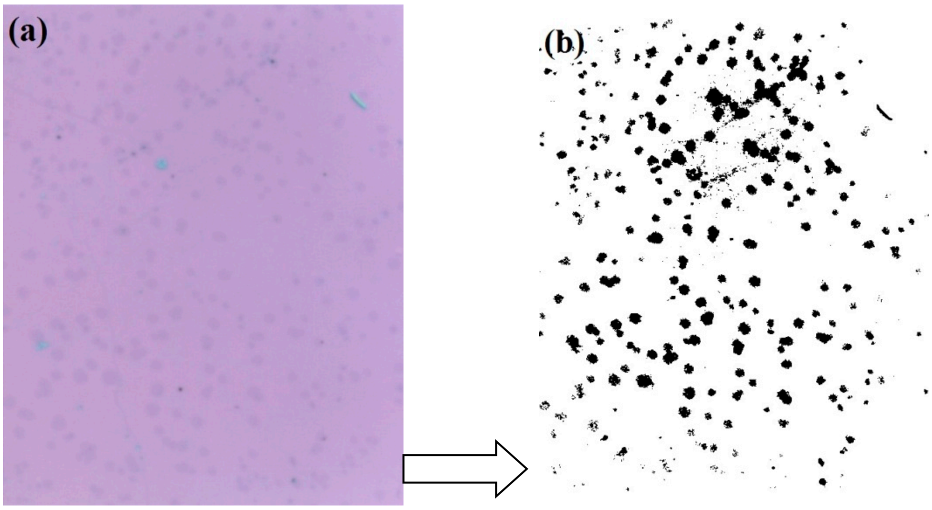

Figure S7. Optical image of graphene film transferred onto $SiO_2$300 nm after synthesis on copper foil with 5 hours hydrogen annealing, nitric acid treatment and electrochemical polishing (**a**). Image translation into two-color format for counting the number of additional graphene layers (**b**). The amount of second graphene layer is 4.0%.
